# Limitation of Water-Soluble Tetrazolium Salt for the Cytocompatibility Evaluation of Zinc-Based Metals

**DOI:** 10.3390/ma14216247

**Published:** 2021-10-20

**Authors:** Peijun Zhu, Jiahao Chen, Ping Li, Shulan Xu

**Affiliations:** Center of Oral Implantology, Stomatological Hospital, Southern Medical University, Guangzhou 510280, China; pjzzpj2255@163.com (P.Z.); graholl@smu.edu.cn (J.C.)

**Keywords:** biodegradable metals, zinc, cytotoxicity, degradation, tetrazolium-based assays

## Abstract

Zinc (Zn) and its alloys have been regarded as promising biodegradable metals. The standardized cytotoxicity evaluation is a mandatory step to screen the biocompatibility of novel Zn and its alloys. Nevertheless, the suitability of the tetrazolium-based assay in the direct contact test for some metallic biomaterials (i.e., magnesium and manganese) is questionable. In this study, our results demonstrate an obvious inconsistency between qualitative observation via fluorescence staining and quantitative assessment using water-soluble tetrazolium salt (CCK-8). Subsequent experiments revealed that Zn and pre-treated Zn can directly convert tetrazolium salts to formazan, falsifying the cytotoxicity results. Therefore, we conclude that the CCK-8 assay is not suitable for evaluating the cytotoxicity of biodegradable Zn-based metals in the direct contact test.

## 1. Introduction

Biodegradable metals (BMs) have been considered as promising implant materials especially for oral and maxillofacial applications, such as osteosynthesis implant and guided bone membrane, etc. In principle, BMs refers to metallic materials that degrade safely and gradually within the human body [1,2,3]. Representative BMs are magnesium (Mg), iron (Fe), zinc (Zn) and their alloys. Mg-based implant materials have been widely investigated and even used in clinical trials [1,3]. Admittedly, regarding the issue of Mg biodegradation, the rapid degradation rate accompanied by the accumulation of hydrogen of Mg-based materials could impede tissue healing and remodeling [4,5]. On the contrary, Fe-based implant materials within bony environments exhibit relatively slow degradation behavior, especially for the formation of insoluble degradation products, which also adversely affects tissue remodeling [6,7].

To date, Zn-based BMs have attracted increasing attention and investigation thanks to their superior biocompatibility and moderate degradation behavior [8,9,10]. Undoubtedly, ionic Zn, as the main degradation product of Zn-based metals, is an essential mineral in the human body, dominating functional roles in metabolism, immune system and nervous system [11,12]. Considering standard corrosion potential, the value of Zn (−0.7 V_SCE_) is between those of Mg (−2.3 V_SCE_) and Fe (−0.4 V_SCE_) [13,14]. Previous in vivo studies demonstrated that a Zn-based stent had steady degradation behavior in the arterial environment and no local/systemic toxicity over one year after implantation [15,16]. Nonetheless, pure Zn is not without its issues for clinical applications. The main disadvantage of pure Zn is its relatively poor strength and ductility, making it insufficient for load-bearing applications [9,17]. To overcome this shortcoming, mechanical strength can be enhanced by adding alloying elements, mainly including magnesium, silver, copper (Cu) and manganese (Mn), etc. [12,18,19]. Thereby, various Zn-based alloy systems have been developed and fabricated, while their biocompatibility should be screened and identified.

**Figure 1:**
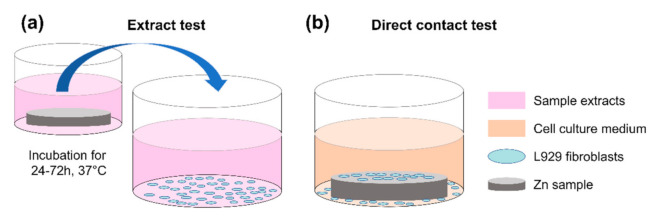
Schematic diagram of test methods for cytotoxicity evaluation of Zn–based BMs: (**a**) extract test and (**b**) direct contact test.

Biocompatibility is a prerequisite requirement for potential Zn-based BMs in clinical use. Considering the ethical issues and minimization of the number of animal tests, a standardized in vitro evaluation system is a critical step to screen and predict the biocompatibility [20]. Based on ISO 10993, various tests methods can be used for the biological evaluation of novel medical biomaterials and devices. Regarding in vitro biocompatibility of biodegradable metallic implants, biological safety can mainly be determined by degradation-related factors, mainly including chemical composition, production residues, degradation products, wear debris, and surface effects [21,22]. Thereby, various biological testing methods in the ISO 10993, such as “genotoxicity” (Part 3), “hemocompatibility” (Part 4), “cytotoxicity” (Part 5), and “systemic toxicity” (Part 11), are required to evaluate the novel Zn-based materials [9,23]. The cytotoxicity assessment method is a relatively simple testing method with the advantages of high replicability, accurate results, and standardized assessment. As illustrated in Figure 1, two main methods for the cytotoxicity evaluation, i.e., an extract test and a direct contact test, can be used as per ISO 10993:5. Among these, the direct contact test is the main method to investigate cellular responses on the material’s surface. Meanwhile, tetrazolium salt-based assays, such as WST-8 (2-(2-methoxy-4-nitrophenyl)-3-(4-nitrophenyl)-5-(2,4-disulfophenyl)-2H-tetrazolium monosodium salt) (commercially available as a CCK-8 reagent) are widely used tools to measure the inhibition of cell metabolic activity. In principle, the tetrazolium salt-based assay is converted by mitochondrial dehydrogenases to a colored compound formazan. The result is produced in a quantity proportional to the number of viable cells. Nonetheless, the results of tetrazolium-based assays can be confounded by the presence of metals, such as Mg, Cu, Mn, leading to false positive or negative results [24,25,26]. To the best of our knowledge, the possible interference between Zn-based metals and tetrazolium-based assays remains unclear.

The aim of this study was to investigate the suitability of a tetrazolium-based assay for the in vitro cytotoxicity evaluation of biodegradable zinc-based materials via a direct contact test. Additionally, potential factors influencing the tetrazolium-based assay were further discussed and clarified.

## 2. Materials and Methods

### 2.1. Specimen Preparation and Surface Characterization

Pure zinc discs (purity 99.99%, 10 mm in diameter, 1 mm in thickness) were used (denoted as Zn). The entire surface of the specimen was wet ground until reaching 1200 grit with silicon carbide abrasive paper. Next, specimens were cleaned ultrasonically in absolute ethanol for 10 min. Pure Zn was further pre-treated with a complete cell culture medium under standard cell culture conditions (denoted as P-Zn). The formation of the initial degradation layer can partially mimic the degradation interface changes occurring in the early post-implantation stage, as previously reported [27,28,29]. Specifically, Zn discs were immersed in 2 mL Dulbecco’s modified Eagle medium (DMEM, Gibco, Grand Island, NY, USA) supplemented with 10% fetal bovine serum (FBS, ExCell Bio, Shanghai, China) under standard cell culture conditions for 7 days. Surface morphology and its elemental composition were characterized by a scanning electron microscope with an energy dispersive X-ray spectroscopy instrument (SEM-EDS, MIRA4 LMH, TESCAN, Brno, Czech Republic). According to ISO 10993-12: 2012 [30], titanium alloy (Ti-6Al-4V) and pure Cu were used as a negative control (N.C.) and positive control (P.C.), respectively. Prior to cell culture experiments, all samples were disinfected with ultraviolet radiation for 1 h.

### 2.2. Direct Contact Test

A direct contact test was used for cytotoxicity evaluation as per ISO 10993-5: 2009 [31]. Additionally, a cell line (L929 mouse fibroblast, Chinese Academy of Sciences, Shanghai, China) was used. Both qualitative and quantitative assessments were performed: cell viability determined by membrane integrity was qualitatively observed by means of live/dead fluorescence staining, and the relative metabolic activity was quantitatively measured using the CCK-8 assay (Dojindo Laboratories Co., Kumamoto, Japan).

L929 fibroblasts were cultured in a DMEM medium with 10% FBS and 1% penicillin-streptomycin (10,000 U/mL penicillin, 10,000 μg/mL streptomycin; Gibco, Paisley, UK). L929 cells were grown under standard cell culture conditions. Additionally, the cell culture medium was renewed two or three times per week. When L929 cells reached approximately 80% confluency, fibroblasts were detached from the flask with 0.25% Trypsin-EDTA (Gibco, Grand Island, NY, USA) to make further passages or carry out the direct contact test.

Regarding the direct contact experiments, specimens were processed in parallel using 12-well culture plates. For each experiment, three parallel samples were used per group. L929 fibroblasts were seeded on the different samples at a density of 3 × 10^4^ cells/cm^2^ for 24 h. The cell membrane integrity was stained by using a fluorescent live/dead viability assay kit (KGAF001, KeyGEN BioTECH, Nanjing, China) with calcein acetoxymethyl (Calcein-AM) reagent and propidium iodide (PI). Samples were stained for 10 min in 10 mL PBS containing 5 μL of 16 mM PI and 5 μL of 4 mM Calcein-AM, following the manufacturer’s instructions. The cell membrane was documented by exemplary photographs using an inverted fluorescence microscope (DMi8, Leica Microsystems GmbH, Wetzlar, Germany). The inhibition of the metabolic activity was quantitatively measured using the CCK-8 assay. To avoid the interference of serum with the tetrazolium-based assay, samples were placed into 48-well plates, and 500 μL fresh medium was added to each well. Afterwards, 50 μL of CCK-8 reagent was added to each sample and for 1 h, following the manufacturers’ instructions. Next, optical density (OD) was measured at 450 nm in a microplate reader (iMark, Bio-Rad, Hercules, CA, USA). To evaluate relative metabolic activity referring to the negative control, the following formula was used (Equation (1)):

Relative metabolic activity (%) = ((OD_test_ − OD_blank_)/(OD_negative_ − OD_blank_)) × 100% (1)

Herein, OD_test_ presents the OD value of the tested specimens. OD_negative_ and OD_blank_ are the OD values of the negative group and group without samples, respectively.

### 2.3. Acellular Assay

To clarify the possible interference between Zn and CCK-8 assays, we explored whether the Zn and P-Zn samples react with the CCK-8 assay. An acellular assay was performed. Samples were incubated with a cell culture medium without cell seeding for 24 h, as mentioned previously (Section 2.2). Afterwards, samples were transferred to 48-well plates, and a fresh medium containing 10% CCK-8 reagent was then added. The OD values were measured as mentioned above.

### 2.4. Statistical Analysis

Based on the results of preliminary experiments, the sample size was calculated for tests using a software program (OpenEpi: Open Source Epidemiologic Statistics for Public Health; www.OpenEpi.com, accessed on 15 October 2021). Thereby, all experiments were performed at least three times. Datasets were further assessed by the Shapiro–Wilk normality test. The results of metabolic activity and the acellular assay were evaluated by using a one-way analysis of variance (ANOVA) with materials as an independent factor, followed by Tukey’s multiple comparisons test. Statistical analyses were performed by GraphPad Prism (Prism 6.01, GraphPad Software, SanDiego, CA, USA), and *p* < 0.05 was regarded as statistically significant.

## 3. Results

### 3.1. Surface Characteristics

**Figure 2:**
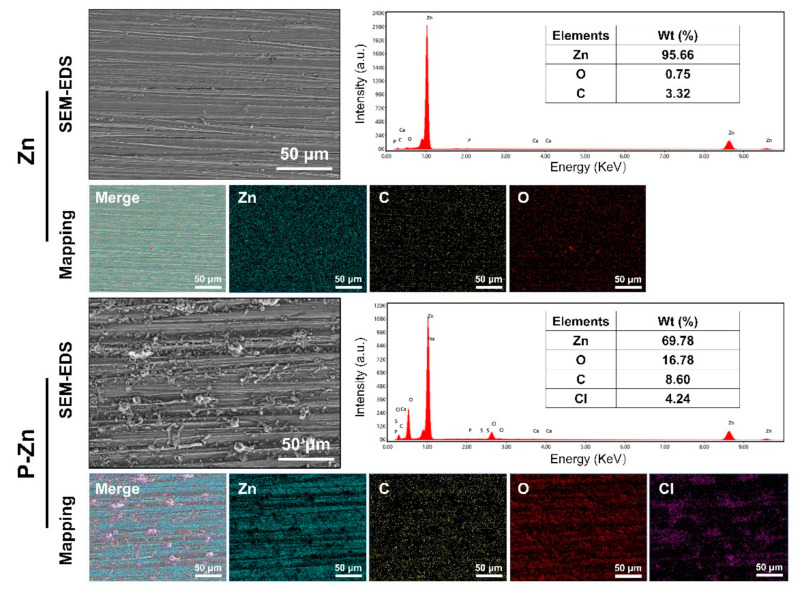
Surface morphology and chemical composition of Zn and P-Zn. Representative SEM images (scale bar = 50 μm, magnification 500×). EDX measurement is indicated by the whole area, showing the elemental composition (energy range between 0–10 keV). EDS mapping showed the elemental distribution.

Surface characterization of the pure Zn and pre-treated Zn by SEM-EDS was revealed, as shown in Figure 2. Relatively uniform parallel scratches can be observed on the Zn surface, while the thin degradation layers were formed on the surface of the pre-treated Zn sample. Regarding the pre-treated Zn sample, EDS analysis indicated that the main elemental components were Zn, C, O and Cl. The results indicate the formation of oxide and carbonate degradation products, which is consistent with previous studies [27,28,29].

### 3.2. Effect of Zn-Based Metals on Cell Membrane Integrity

**Figure 3:**
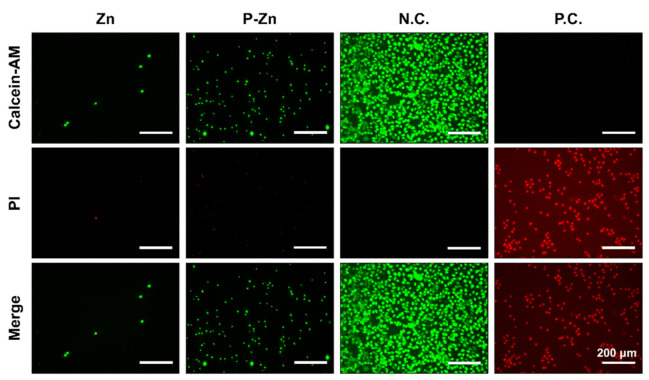
Representative fluorescent live/dead staining of L929 fibroblasts cultured on sample surfaces for 24 h. A titanium-based alloy was used as negative control (N. C.) and pure copper was used as a positive control (P. C.). Green fluorescence represents living fibroblasts stained with calcein-AM, and red fluorescence represents apoptotic cells with compromised membrane integrity, stained with PI. (magnification 100×; scale bar = 200 μm).

The potential impact on cell membrane damage was qualitatively determined by live/dead staining. Figure 3 shows that only a few L929 fibroblasts on the original Zn surface exhibited green fluorescence, implying mostly apoptotic cells. In addition, more L929 cells on the pre-corroded Zn surface showed predominantly irregular-shaped morphologies with intact outlines, indicating that cytotoxic effects reduced after pre-treatment. However, the number of viable cells on P-Zn surfaces was significantly lower compared to the counterpart of the negative control.

### 3.3. Influence of Zn-Based Metals on Cell Metabolic Activity

**Figure 4:**
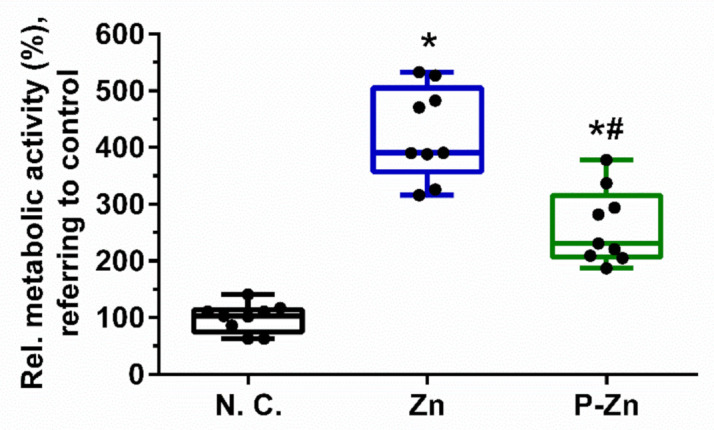
Relative metabolic activity of L929 cells cultured on titanium, zinc and pre-treated zinc for 24 h, measured by CCK-8 assay. A titanium-based alloy was used as a negative control and set to 100%. The box-and-whisker plot indicates the combined results from three independent experiments with three samples per group in each run. Black dots represent each tested sample (*n* = 9). * and # represent *p* < 0.05 when compared to the negative control and pure Zn, respectively.

Impairment of cell metabolic activity was determined by CCK-8 assay, as shown in Figure 4. One-way ANOVA showed significant differences in relative cell metabolic activity among different materials (F (2, 24) = 61.49, *p* < 0.0001). Furthermore, Tukey’s multiple comparisons showed that the cell metabolic activity of the negative control group (99.67 ± 25.39) was significantly lower than those of Zn (425.0 ± 81.38, *p* < 0.0001) and P-Zn (260.4 ± 65.96, *p* < 0.0001), respectively. This indicated that viable L929 cells were grown on the surfaces of Zn and P-Zn. Nevertheless, our results show obviously inconsistent results between qualitative observation via live/dead staining and quantitative assessment by CCK-8 assay.

### 3.4. Role of Zn-Based Metals in Formazan Formation

**Figure 5:**
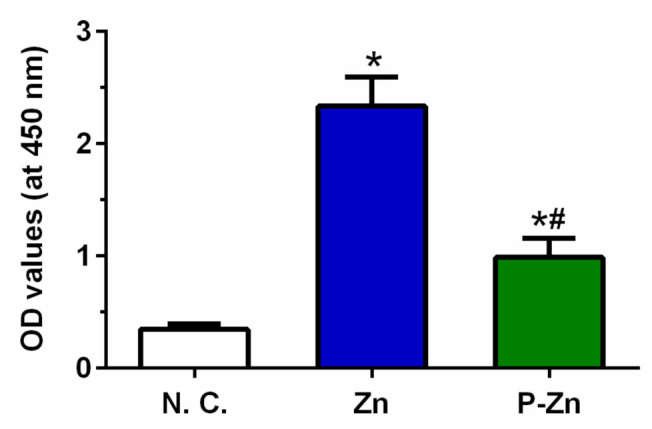
The acellular assay indicates that the OD value is influenced by Zn and pretreated Zn alloys. Fresh medium with 10% CCK-8 reagent was added to samples without cells for 1 h and the values of optical density were read at 450 nm. The chart shows the combined results from three independent experiments with three samples per group in each run. Each bar represents means and standard deviation (*n* = 9). * and # represent *p* < 0.05 when compared to the negative control and pure Zn, respectively.

To explore the interfere with Zn-based material and CCK-8 reagent, the acellular experiment was performed. As shown in Figure 5, the results reveal that the OD values of Zn and P-Zn were significantly higher than that of the negative control (*p* < 0.0001), determined by one-way ANOVA. Therefore, the Zn-based metallic materials caused obvious interference with the formazan of the CCK-8 assay.

## 4. Discussion

Since the development of novel Zn-based BMs, questions about in vitro cytotoxicity evaluation have arisen. The tetrazolium-based cytotoxicity assay is mostly used to assess cell metabolic activity directly. Nevertheless, water-soluble tetrazolium salt can be interfered with by Mg-, Mn- or Cu-containing biomaterials, leading to false negative/positive cytotoxicity results. In this study, the CCK-8 assay was evaluated with respect to its suitability for Zn-based metallic materials. Our results demonstrate that Zn-based metals interfere with the reaction of CCK-8 reagent and generate false negative cytotoxicity results.

Admittedly, the link between in vitro and in vivo biological evaluation for Zn-based materials is relatively poor. Previously, numerous in vivo studies demonstrated that Zn-based implant materials had no apparent adverse effects on local and systemic tissues, indicating excellent biocompatibility [32,33]. Nonetheless, Zn and Zn-based alloys showed obvious cytotoxicity, especially for the direct contact test. In principle, the standardized direct contact test aims to investigate the cellular behavior on the material. The cytotoxicity results are mainly determined by surface characteristics as well as the degradation products released.

In our tests, the cytotoxicity is directly linked to Zn degradation. Firstly, L929 fibroblasts were nonviable on the bare Zn surfaces (Figure 3), in line with most previous studies [34,35,36]. High concentration and rapid release of Zn ions could lead to this cytotoxic effect [36]. Specifically, when samples were immersed in the cell culture medium, the coupled anodic dissolution and the cathodic reduction will take place progressively, as described in Equations (2) and (3) [8,37,38]. In addition, L929 cells can grow directly on the pre-treated Zn alloy surface due to the formation of passivation layers (Figure 2). According to the Pourbaix diagram, zinc hydroxide (Zn(OH)_2_) and zincite (ZnO) can be formed on the surface as the passivation layers, as described in Equations (4) and (5). The passivation layers can effectively decrease Zn ion concentration released from Zn-based BMs, which is in line with the previous investigations [28,35].

Anodic reaction: Zn (s) → Zn^2+^ (aq) + 2e^−^ (2)

Cathodic reaction: 2H_2_O (l) + O_2_ (aq)+ 4e^−^ (aq) → 4OH^−^ (aq) (3)

Zn(OH)_2_ formation reaction: Zn^2+^ (aq)+ 2OH^−^ (aq) → Zn(OH)_2_ (s) (4)

ZnO formation reaction: Zn^2+^ (aq) + 2OH^−^ (aq) → ZnO (s) + H_2_O (l) (5)

The inconsistent results were observed between qualitative observations (Figure 3) and quantitative assessment (Figure 4). To verify the results, the acellular assay was performed (Figure 5). Regarding the samples without cells, a formazan dye was also observed, demonstrating the interference of Zn-based materials with the CCK-8 reagent.

**Figure 6:**
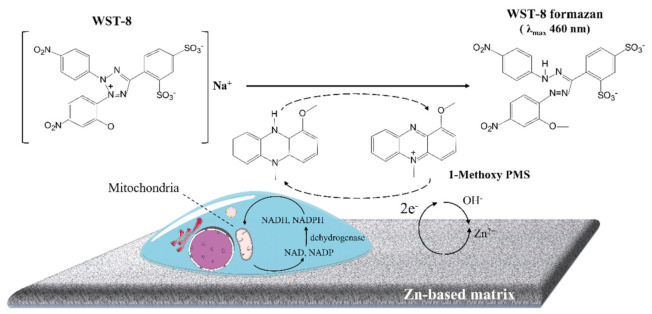
Schematic diagram of the principle of the metabolic activity detected by the CCK−8 assay and the assumed formation of formazan by Zn−based metals.

In principle, the CCK-8 reagent contains WST-8 and 1-methoxy-5-methylphenazinium methyl sulfate (1-methoxy PMS) to evaluate the metabolic activity of viable cells. As illustrated in Figure 6, the tetrazolium-based salt, WST-8, can be reduced via a reaction with the reduced form of 1-methoxy PMS. Specifically, the mediator directly reacts with reduced nicotinamide adenine dinucleotide (NADH) and nicotinamide adenine dinucleotide phosphate (NADPH). Meanwhile, the reaction of dehydrogenase enzymes and their substrates, e.g., lactate dehydrogenase and lactic acid, triggers NAD or NADP to NADH and NADPH. Thereby, the tetrazolium salt is utilized to determine the dehydrogenase activity or a substrate of the dehydrogenase [39].

In our acellular experiments, the Zn and pre-treated Zn interfere with the formazan of CCK-8 assay (Figure 5). As shown in Figure 6, we supposed that Zn-based metals, as a reducing agent, have a strong tendency to lose electrons due to the anodic reaction. The electron-transport mediator (1-Methoxy PMS) receives these electrons from Zn and passes the electron to form the WST-8 formazan. Thus, Zn-based metals can lead to false-negative cytotoxicity results.

Notably, previous cytotoxicity tests reported that the CCK-8 reagent was used to analyze cell viability and proliferation directly grown on Zn or Zn-based metallic materials [40,41]. Nevertheless, based on our results, the suitability of the CCK-8 assay in the direct contact test appears to be questionable. Meanwhile, a similar phenomenon may occur in other tetrazolium-based assays, i.e., 3-(4,5-dimethylthiazol-2-yl)-2,5-diphenyl tetrazolium bromide (MTT). It is of utmost importance to carefully check for interference in the tetrazolium-based assay and to rely on less error-prone assays, such as DNA measurement (bromodeoxyuridine incorporation assay). Therefore, our results demonstrate for the first time that biodegradable Zn-based metals interfere with the formazan of WST-8 and generate a false-negative cytotoxicity result, probably due to the anodic reaction of Zn.

## 5. Conclusions

Zn and its alloys are considered as promising absorbable biomaterials for temporary implants such as osteosynthesis implants and vascular stents. The in vitro cytotoxicity tests are essential methods to evaluate the potentially toxic effects in vivo. Tetrazolium-based assays such as CCK-8 are frequently used to determine cell viability due to their precision, sensitivity and easy handling. Nevertheless, our results demonstrate that direct cells cultured on Zn-based surfaces lead to apparent misleading cytotoxicity with the CCK-8 assay. Further studies are required to identify appropriate methods for the assessment of Zn cytotoxicity.

## Data Availability

The data presented in this study are available on request from the corresponding author.

## References

[1] Han H.-S., Loffredo S., Jun I., Edwards J., Kim Y.-C., Seok H.-K., Witte F., Mantovani D., Glyn-Jones S. (2019). Current status and outlook on the clinical translation of biodegradable metals. Mater. Today.

[2] Liu Y., Zheng Y., Chen X.-H., Yang J.-A., Pan H., Chen D., Wang L., Zhang J., Zhu D., Wu S. (2019). Fundamental Theory of Biodegradable Metals-Definition, Criteria, and Design. Adv. Funct. Mater..

[3] Zhao D., Witte F., Lu F., Wang J., Li J., Qin L. (2017). Current status on clinical applications of magnesium-based orthopaedic implants: A review from clinical translational perspective. Biomaterials.

[4] Kraus T., Fischerauer S.F., Hänzi A.C., Uggowitzer P.J., Löffler J.F., Weinberg A.M. (2012). Magnesium alloys for temporary implants in osteosynthesis: In vivo studies of their degradation and interaction with bone. Acta Biomater..

[5] Geis-Gerstorfer J., Schille C., Schweizer E., Rupp F., Scheideler L., Reichel H.P., Hort N., Nolte A., Wendel H.P. (2011). Blood triggered corrosion of magnesium alloys. Mater. Sci. Eng. B.

[6] Pierson D., Edick J., Tauscher A., Pokorney E., Bowen P., Gelbaugh J., Stinson J., Getty H., Lee C.H., Drelich J. (2012). A simplified in vivo approach for evaluating the bioabsorbable behavior of candidate stent materials. J. Biomed. Mater. Res. Part B.

[7] Drynda A., Hassel T., Bach F.W., Peuster M. (2015). In vitro and in vivo corrosion properties of new iron-manganese alloys designed for cardiovascular applications. J. Biomed. Mater. Res. Part B.

[8] Levy G.K., Goldman J., Aghion E. (2017). The Prospects of Zinc as a Structural Material for Biodegradable Implants-A Review Paper. Metals.

[9] Zhang W., Li P., Neumann B., Haag H., Li M., Xu Z., Zhou C., Scheideler L., Wendel H.P., Zhang H. (2021). Chandler-Loop surveyed blood compatibility and dynamic blood triggered degradation behavior of Zn-4Cu alloy and Zn. Mater. Sci. Eng. C.

[10] Hosova K., Pinc J., Skolakova A., Bartunek V., Vertat P., Skolakova T., Prusa F., Vojtech D., Capek J. (2021). Influence of Ceramic Particles Character on Resulted Properties of Zinc-Hydroxyapatite/Monetite Composites. Metals.

[11] Su Y., Cockerill I., Wang Y., Qin Y.X., Chang L., Zheng Y., Zhu D. (2019). Zinc-Based Biomaterials for Regeneration and Therapy. Trends Biotechnol..

[12] Li P., Schille C., Schweizer E., Kimmerle-Müller E., Rupp F., Han X., Heiss A., Richter A., Legner C., Klotz U.E. (2019). Evaluation of a Zn-2Ag-1.8Au-0.2V Alloy for Absorbable Biocompatible Materials. Materials.

[13] Eliaz N. (2019). Corrosion of Metallic Biomaterials: A Review. Materials.

[14] Avior O., Ben Ghedalia-Peled N., Ron T., Vago R., Aghion E. (2020). The Effect of Ca on In Vitro Behavior of Biodegradable Zn-Fe Alloy in Simulated Physiological Environments. Metals.

[15] Drelich A.J., Zhao S., Guillory R.J., Drelich J.W., Goldman J. (2017). Long-term surveillance of zinc implant in murine artery: Surprisingly steady biocorrosion rate. Acta Biomater..

[16] Yang H., Wang C., Liu C., Chen H., Wu Y., Han J., Jia Z., Lin W., Zhang D., Li W. (2017). Evolution of the degradation mechanism of pure zinc stent in the one-year study of rabbit abdominal aorta model. Biomaterials.

[17] Li P., Zhang W., Dai J., Xepapadeas A.B., Schweizer E., Alexander D., Scheideler L., Zhou C., Zhang H., Wan G. (2019). Investigation of zinc-copper alloys as potential materials for craniomaxillofacial osteosynthesis implants. Mater. Sci. Eng. C.

[18] Li P., Schille C., Schweizer E., Rupp F., Heiss A., Legner C., Klotz U.E., Geis-Gerstorfer J., Scheideler L. (2018). Mechanical Characteristics, In Vitro Degradation, Cytotoxicity, and Antibacterial Evaluation of Zn-4.0Ag Alloy as a Biodegradable Material. Int. J. Mol. Sci..

[19] Čapek J., Kubásek J., Pinc J., Fojt J., Krajewski S., Rupp F., Li P. (2021). Microstructural, mechanical, in vitro corrosion and biological characterization of an extruded Zn-0.8Mg-0.2Sr (wt%) as an absorbable material. Mater. Sci. Eng. C.

[20] Li P., Schille C., Schweizer E., Kimmerle-Müller E., Rupp F., Heiss A., Legner C., Klotz U.E., Geis-Gerstorfer J., Scheideler L. (2019). Selection of extraction medium influences cytotoxicity of zinc and its alloys. Acta Biomater..

[21] Loos A. (2015). Biocompatibility Testing and Marketing Authorisation of Degradable Magnesium Implants, Surface Modification of Magnesium and Its Alloys for Biomedical Applications.

[22] Willbold E., Weizbauer A., Loos A., Seitz J.M., Angrisani N., Windhagen H., Reifenrath J. (2017). Magnesium alloys: A stony pathway from intensive research to clinical reality. Different test methods and approval—Related considerations. J. Biomed. Mater. Res. A.

[23] Li P., Zhang W., Spintzyk S., Schweizer E., Krajewski S., Alexander D., Dai J., Xu S., Wan G., Rupp F. (2021). Impact of sterilization treatments on biodegradability and cytocompatibility of zinc-based implant materials. Mater. Sci. Eng. C..

[24] Semisch A., Hartwig A. (2014). Copper ions interfere with the reduction of the water-soluble tetrazolium salt-8. Chem. Res. Toxicol..

[25] Scarcello E., Lambremont A., Vanbever R., Jacques P.J., Lison D. (2020). Mind your assays: Misleading cytotoxicity with the WST-1 assay in the presence of manganese. PLoS ONE.

[26] Al Hegy A., Smith R., Gauthier E.R., Gray-Munro J.E. (2020). Investigation of a cyanine dye assay for the evaluation of the biocompatibility of magnesium alloys by direct and indirect methods. Bioact. Mater..

[27] Fu J., Su Y., Qin Y.X., Zheng Y., Wang Y., Zhu D. (2020). Evolution of metallic cardiovascular stent materials: A comparative study among stainless steel, magnesium and zinc. Biomaterials.

[28] Jablonská E., Vojtěch D., Fousová M., Kubásek J., Lipov J., Fojt J., Ruml T. (2016). Influence of surface pre-treatment on the cytocompatibility of a novel biodegradable ZnMg alloy. Mater. Sci. Eng. C.

[29] Levy G.K., Kafri A., Ventura Y., Leon A., Vago R., Goldman J., Aghion E. (2019). Surface stabilization treatment enhances initial cell viability and adhesion for biodegradable zinc alloys. Mater. Lett..

[30] ISO 10993-12: 2012 (2012). Biological Evaluation of Medical Devices—Part 12: Sample Preparation and Reference Materials.

[31] ISO 10993-5: 2009 (2009). Biological Evaluation of Medical Devices—Part 5: Tests for In Vitro Cytotoxicity.

[32] Guo H., Xia D., Zheng Y., Zhu Y., Liu Y., Zhou Y. (2020). A pure zinc membrane with degradability and osteogenesis promotion for guided bone regeneration: In vitro and in vivo studies. Acta Biomater..

[33] Yang H., Jia B., Zhang Z., Qu X., Li G., Lin W., Zhu D., Dai K., Zheng Y. (2020). Alloying design of biodegradable zinc as promising bone implants for load-bearing applications. Nat. Commun..

[34] Wang C., Yang H.T., Li X., Zheng Y.F. (2016). In Vitro Evaluation of the Feasibility of Commercial Zn Alloys as Biodegradable Metals. J. Mater. Sci. Technol..

[35] Shearier E.R., Bowen P.K., He W., Drelich A., Drelich J., Goldman J., Zhao F. (2016). In Vitro Cytotoxicity, Adhesion, and Proliferation of Human Vascular Cells Exposed to Zinc. ACS Biomater. Sci. Eng..

[36] Su Y., Wang K., Gao J., Yang Y., Qin Y.X., Zheng Y., Zhu D. (2019). Enhanced cytocompatibility and antibacterial property of zinc phosphate coating on biodegradable zinc materials. Acta Biomater..

[37] Chen Y., Zhang W., Maitz M.F., Chen M., Zhang H., Mao J., Zhao Y., Huang N., Wan G. (2016). Comparative corrosion behavior of Zn with Fe and Mg in the course of immersion degradation in phosphate buffered saline. Corros. Sci..

[38] Bowen P.K., Drelich J., Goldman J. (2013). Zinc exhibits ideal physiological corrosion behavior for bioabsorbable stents. Adv. Mater..

[39] Held P. (2009). An Absorbance-Based Cytotoxicity Assay Using High Absorptivity, Water-Soluble Tetrazolium Salts, Application Note.

[40] Yang Y., Cheng Y., Peng S., Xu L., He C., Qi F., Zhao M., Shuai C. (2021). Microstructure evolution and texture tailoring of reduced graphene oxide reinforced Zn scaffold. Bioact. Mater..

[41] Lin J., Tong X., Shi Z., Zhang D., Wen C. (2020). A biodegradable Zn-1Cu-0.1Ti alloy with antibacterial properties for orthopedic applications. Acta Biomater..

